# Heart failure patients’ experiences of self-care neglect: a content analysis

**DOI:** 10.1186/s12872-024-04347-3

**Published:** 2024-12-20

**Authors:** Parisa Sadat Bahrodi, Azade Safa, Neda Mirbagher Ajorpaz, Fatemeh Sadat Izadi Avanji

**Affiliations:** 1 Trauma Nursing Research Center, Kashan University of Medical Sciences , Kashan, Iran; 2 Autoimmune Diseases Research Center, Kashan University of Medical Sciences, Kashan, Iran

**Keywords:** Heart failure, Neglect, Qualitative content analysis, Self-care

## Abstract

**Background:**

Heart failure is a chronic and common disorder worldwide. Patients with heart failure need self-care behaviors to manage their condition. Despite the importance of self-care in positive health outcomes, many patients with heart failure neglect their self-care behaviors. Therefore, the present study was conducted to explain heart failure patients’ experiences of self-care neglect.

**Methods:**

This qualitative study was conducted using conventional content analysis method. Participants included 15 patients with heart failure. Data were collected through semi-structured interviews and using purposive sampling method. Sampling continued until data saturation was reached. Data analysis was performed concurrently with data collection. Lincoln and Guba’s four criteria were used to ensure the trustworthiness of the data. Data management was performed using MAXQDA version 24 software.

**Results:**

The results were presented in the form of four main categories and 10 subcategories. In analyzing the data of the study, four main categories emerged in the participants’ experiences: “false cultural beliefs in self-care”, “weakening of mental-psychological power”, “synergy of physical problems”, and “inappropriate support”.

**Conclusion:**

The patients in their experiences of neglect in self-care pointed to false cultural beliefs in self-care, weakening of mental-psychological power, synergy of physical problems, and inappropriate support. Knowing the factors that influence self-care neglect and preventing their occurrence can improve self-care skills and prevent neglect-related side effects in patients with heart failure. Healthcare providers can help improve the health of these patients by developing interventions to mitigate these factors. It is suggested that future research be designed in the form of an intervention to reduce the effect of each of these factors.

**Trial registration:**

This is a qualitative study and has not been registered in Iranian Registry of Clinical Trials.

## Background

Heart failure (HF) is a chronic and prevalent disorder in which the heart loses its ability to pump blood necessary for the body’s metabolic needs [1]. It affects more than 37 million people worldwide [2]. Cardiovascular diseases are the leading cause of death in Iran [3]. This disease leads to activity intolerance, disruption of social interactions, psychological distress, decreased vitality, increased dependency, early retirement and lifestyle changes in the patient, which in turn can reduce patient satisfaction and quality of life and impose high costs on the patient, family and society [4]. Therefore, these patients need self-care behaviors to manage the problems associated with the disease.

The concept of self-care was first proposed by Dorothea Orem in 1959 as Orem’s Self-Care Theory. Orem defines self-care as the actions a person takes to maintain or improve his or her life, health, well-being, and the prevention and treatment of illness [5]. Self-care includes three key principles of self-care maintenance, self-care management, and symptom recognition [6]. Heart failure self-care is a set of behaviors, including diet and medications, limiting sodium and fluid intake, the amount of activity allowed, weighing oneself daily, and seeking and deciding on appropriate treatment if the condition worsens. In fact, the most important principle of self-care is the patient’s participation and taking responsibility in the treatment process, so that many of the complications of the disease can be controlled by performing the appropriate behaviors [7, 8]. Therefore, more effective self-care behaviors will improve patients’ quality of life and reduce mortality and re-hospitalization rates [9].

Despite the prominent role of self-care behaviors in preventing the serious consequences of heart failure, evidence shows that self-care is weak in these patients. In this regard, the results of Jaarsma’s study showed that most patients with heart failure had poor self-care behaviors and were negligent in their self-care [10]. In Iran, Asadi et al. (2019) found in a study that 67% of the patients with heart failure had moderate self-care behaviors, which led to poor health outcomes and their re-hospitalization [11]. The results of another study conducted in Iran showed that only 5% of the patients with heart failure had good self-care behaviors (9). In another study, at least 50% of patients with heart failure did not follow their treatment recommendations, which increased their re-hospitalization [12]. Despite the importance of self-care in positive health outcomes, many patients with heart failure neglect their self-care behaviors. This may be due to the complexity of self-care, lack of perceived need for self-care, long-term behavior change, or lack of motivation [13].

Negligence in the Oxford dictionary means failure to give somebody/something enough care or attention [14]. Self-neglect is a person’s failure to take necessary care in self-care. It manifests itself in the form of non-adherence to treatment, failure to perform health behaviors, and inconsistent health behaviors [15]. Therefore, any neglect of self-care in terms of personal, health, hygiene, and living conditions can be called self-neglect. Neglect of self-care in chronic disease worsens disease and disrupts quality of life [16]. Day et al. (2015) define self-care neglect as a person’s failure to meet their basic needs [17]. Most cases of exacerbation of heart failure are due to self-care neglect in these patients [18]. Self-care neglect can be influenced by cultural and social factors. Cultural, economic, and social developments in society in recent years, especially with the growth of health-related technologies, may also affect the self-care of patients with heart failure [19]. Healthcare providers should increase their knowledge of the unknown factors in the area of self-care neglect in these patients, so that they can improve the health level of the patients and help them perform self-care behaviors.

Although an emphasis has been made on self-care in chronic patients, particularly those with heart failure, a limited number of studies have been conducted on self-care neglect in these patients. In addition, most of the studies conducted in this area have been quantitative and have focused primarily on investigating self-care behaviors and related factors [20, 21]. However, it should be noted that quantitative studies alone cannot lead to the recognition of views, individual needs and understanding of the factors that influence self-care neglect [22]. Therefore, a more comprehensive approach is needed to examine the views of heart failure patients, who have been living with the disease for a long time, in the area of self-care neglect and related challenges. Self-care neglect is a mental phenomenon and a human experience that should be expressed by patients who have understood it [8]. Given the nature of the research objective, a qualitative approach, involving human interpretation and experience, was used in this study. Considering the significance of the problem and the lack of information in this area, the present study was conducted to explain the heart failure patients’ experiences of self-care neglect. Knowing the results of this study is important for healthcare professionals because it can lead to more appropriate interventions, better health outcomes, and improved quality of life for patients.

Considering the significance of the problem and the lack of information in this area, the present study was conducted to explain heart failure patients’ experiences of self-care neglect. Knowing the results of this study is important for healthcare professionals because it can lead to more appropriate interventions, better health outcomes, and improved quality of life for patients.

## Method

### Study design and participants

Using conventional content analysis, this qualitative study was conducted from August 2023 to March 2024 to explain the experiences of heart failure patients with self-care neglect. In this method, based on the explanation of the research participants, the obvious and hidden concepts in their minds are identified, coded, summarized and classified, and the themes are extracted from them [23].

The participants in this study were patients with class 1 to 4 heart failure who were referred to the cardiac departments of governmental and non-governmental hospitals in Kashan and Tehran Heart Center as outpatients or for hospitalization. Inclusion criteria of the participants were diagnosis of heart failure by a cardiologist, awareness of the diagnosis of their disease, physical ability to participate in the study, ability to communicate verbally, age over 18 years, the passage of six months from the start of the treatment, no cognitive problems based on the 10-question AMT test (getting a score of 8 and above), and engaging patients with different characteristics (of both sexes, of different age groups, in different classes of heart failure, different economic and social conditions) [24]. Exclusion criteria included unwillingness to continue in the study or decision to withdraw from the study due to deterioration in the patient’s physical or mental condition.

### Data collection

After approving the research plan and obtaining the code of ethics from Kashan University of Medical Sciences, the researcher proceeded with data collection. The data collection method was semi-structured individual interviews lasting between 40 and 55 min. Purposive sampling was used in this study to obtain rich information about the purpose of the study. The first participant was purposively selected in consultation with hospital nurses from among the heart failure patients with inclusion criteria. Then, after analyzing the data from the interview with the first participant, the next participant was selected. Before the interview began, participants were informed of the purpose of the study and the confidentiality of the information, and their written informed consent was obtained. The interviews were conducted 1–2 times according to the participant’s wish and mental and physical condition. All interviews were recorded using a digital audio recorder. The interviews were conducted by prior arrangement with the participants. The time and place of the interview was determined by taking into account the willingness of the participants. A total of 15 patients were interviewed. In coordination with the participants, 9 interviews were conducted at the participant’s home and 6 interviews at the hospital. One of the participants was also interviewed twice, and the second follow-up interview was conducted by telephone. All interviews were conducted in a quiet and comfortable environment so that the patient could answer the questions with the necessary concentration. Before conducting the interview, the researcher would prepare the ground for better interviews by asking a series of preliminary questions, establishing proper communication, and gaining the trust of the participants. The interview questions are included in the interview guide (Table 1).

**Table 1:** Interview guide

Number	The interview questions
1	Please describe your experience of self-care in heart failure?
2	Explain the areas in which you do not take self-care seriously?
3	What factors contribute to your self-care neglect?
4	Follow-up questions: • What did you mean when you said that? • Please explain more • Please give an example

In order to observe the maximum diversity in sampling, an attempt was made from the beginning of the study to include patients with different characteristics. Data saturation was the criterion for sample size determination. Data saturation occurs when no new data contributing to the definition of the characteristics of a class are included in the research and all desired comparisons have been made [23].

### Data analysis

Data analysis was conducted after the first interview, concurrent with data collection. Data analysis was based on conventional content analysis using Zhang’s 8-step approach [25]:

In the first step, immediately after the end of each interview, the content of the interview was typed verbatim into the Word program and entered into the MAXQDA software version 24 for ease of data management. The unit of analysis refers to the main unit of the written text that needs to be categorized during content analysis. In this step, the written interviews were read several times by the researcher in order to understand the nature of what was being expressed in the raw data. The interview text was then broken down and coded using the participants’ language or words the researcher understood. In the third step, the researcher distinguished between the levels of concepts obtained and began to develop concepts through comparative analysis, questioning, and other analytical methods. In the fourth step, the coding test was performed based on the sample text. At this stage, the researcher provided the typed text of the interview along with the extracted codes to her supervising professor, advisor, and two members of the nursing faculty to check the stability of the coding, and the coding process and the researcher’s interpretation of the participants’ statements were approved by the experts. In the fifth step, the researcher continuously checked the coding during the coding process to ensure that there was agreement between the extracted codes based on the researcher’s inference and the opinion of the study participants and the research team. In the sixth step, the stability of the codes was checked again. Code checking is an ongoing process during content analysis. The seventh step was related to the extraction of results from the coded data and creation categories. Codes that were conceptually similar were placed around a category, and the primary categories were gradually identified. Categories were then developed based on their characteristics and dimensions, similar categories were combined, and more abstract categories and themes were revealed with careful consideration. At this stage, inter-category relationships were identified and hidden and obvious concepts were reviewed. The eighth step was to fully and honestly report the analysis method and work processes, such as coding and the methods used.

### Trustworthiness

To ensure the trustworthiness of the data, Guba and Lincoln’s four criteria (credibility, dependability, transferability and confirmability) were used [26]. Participant verification was used to ensure data credibility. For this purpose, a portion of the interview text along with the initial codes was given to four literate participants to check the compatibility of the findings with their experiences and to correct the concepts that did not reflect their views. The long-term involvement of the researcher with the data also contributed to the credibility of the study. To assess the dependability of the data, a portion of the interviews was given to an external observer who checked and confirmed the extracted concepts. Throughout the research process, the research team had regular meetings for evaluation and approval of the research process. To ensure the transferability of the data, an attempt was made to include patients with different characteristics (of both sexes, of different age groups, in different classes of heart failure, different economic and social conditions). In addition, the researcher gave the results of the study to patients who met the inclusion criteria but did not participate in the study to determine the extent to which the research findings matched their experiences. To ensure the confirmability of the data, the researcher carefully recorded the study process and the decisions made during the research.

### Ethical considerations

The present study was approved under the ethics code IR.KAUMS.NUHEPM.REC.1402.034 by the ethics committee of Kashan University of Medical Sciences. After explaining the purpose of the research and data collection method, written informed consent was obtained from the participants for each interview. They were also assured that the audio files of the interviews, as well as the transcribed texts, would be kept anonymous and confidential only to the researchers, and that the participants’ information would be used with their pseudonyms. They would also be provided with the results of the research if they wished.

## Results

The results of this study were obtained by interviewing 15 heart failure patients. The mean age of the participants was 66.06 years. Of the participants, 8 were women (53.3%) and 7 were men (46.7%). In terms of disease severity, 2 participants (13.3%) had class 1 heart failure, 4 participants (26.7%) class 2, 6 participants (40%) class 3, and 3 participants (20%) class 4. Other characteristics of the participants are shown in Table 2.

**Table 2:** Demographic characteristics of the participants

Row	Sex	Age	Education	Job	Marital status	No. of children	Interview duration (min)	Disease class	No. of interviews
1	Male	44	Bachelor’s degree	Hospital Facilities Coordinator	Married	1	45	4	2
2	Female	79	Secondary education	Housewife	Married	4	60	3	1
3	Female	38	Bachelor’s degree	Housewife	Married	1	65	Congenital/ 3	1
4	Male	83	Elementary	Retired	Widower	1	60	4	1
5	Male	72	Bachelor’s degree	Lawyer	Widower	3	50	2	1
6	Male	72	Uneducated	Rancher	Married	7	40	3	1
7	Male	80	Elementary	Tailor	Married	5	43	2	1
8	Female	68	Elementary	Housewife	Married	3	52	3	1
9	Male	60	Bachelor’s degree	Retired	Married	3	47	1	1
10	Female	82	Uneducated	Housewife	Married	5	45	2	1
11	Male	76	Elementary	Retired	Married	6	52	3	1
12	Female	48	Bachelor’s degree	Teacher	Married	3	56	4	1
13	Female	63	Diploma	Doctor’s secretary	Married	3	41	3	1
14	Female	61	Diploma	Retired	Married	3	40	1	1
15	Female	65	Elementary	---	Widow	2	52	2	1

The results were extracted in the form of 4 main categories (“false cultural beliefs in self-care”, “weakening of mental-psychological power”, “synergy of physical problems”, and “inappropriate support”) and 10 subcategories (Table 3).

**Table 3:** Categories extracted from the experiences of people with heart failure regarding the causes of self-care neglect

Categories	Subcategories	Examples of primary coding
False cultural beliefs in self-care	Put family before self	- Being too busy with chores and not having time for yourself - Prioritization of child care - Prioritization of spouse care
	Belief in the usefulness of non-scientific traditional methods	- Use of traditional medicine to open heart vessels - Use of opium to lower blood sugar - Use of opium to suppress the symptoms
	The taboo of expressing sexual problems caused by the disease	- Lack of education about sexual problems - Lack of sex education out of shame or embarrassment - Shame about expressing sexual problems after illness
weakening of mental-psychological power	Disappointment in the treatment process	- Getting fed up with life in the process of congenital disease treatment - Decreased life expectancy - Despair caused by the difficulties of the disease
	Fear of social stigma	- Embarrassment about using a walker in front of people - Fear of being judged by others - Eating fatty foods at parties for fear of others’ judgment
	Forgetting God	- Laxity of care when the remembrance of God is neglected - Reduced motivation for self-care due to neglect of prayer - Neglected care for blasphemy caused by the problems of illness
Synergy of physical problems	Inability to stop bad eating habits	- Adding salt to food - Drinking a lot of tea per day -Use of animal fat instead of vegetable oil
	Problems associated with comorbidities	- Forgetting to take some pills due to multiple medications - Lack of movement due to knee pain - Mistakenly taking medications due to their similar appearance
Inappropriate support	Insufficient family support	- Spouse smoking at home - Not exercising due to lack of family support - Forgetting to remind mother to take medication
	Insufficient government support	- Delay in visiting a doctor due to the high cost of treatment - Financial problems to purchase medicines - Failure to follow up with nutrition counseling due to lack of free counseling centers - Absence of necessary facilities for periodic examination of the patient

### False cultural beliefs in self-care

False cultural beliefs in this study refer to the specific views and opinions held by people that may negatively influence their health-related behaviors and self-care. This category included the three subcategories of putting family before self, belief in the usefulness of non-scientific traditional methods, and the taboo of expressing sexual problems caused by the disease.

#### Putting family before self

Based on the experiences of most of the participants, it was found that they always prioritize family care in all matters. As a result, they sometimes neglect their own care, especially in relation to their illness. This issue was particularly evident in the words of the female participants, who played a greater role in caring for children and the household.

*When I wake up in the morning*,* I’m busy with kitchen and cleaning stuff; then I deal with the kids. In the evening*,* I catch an hour of sleep if possible*,* and then I’m back to taking care of the kids and my husband. If I’ve some extra time*,* I’ll chill with my phone or watch TV. It’s all about serving the family (with a smile)* (P 3).

*Honestly*,* my kids are my top priority. My hubby is important too. I focus on looking after them rather than myself. Sometimes when I’m feeling down*,* I’ll just go to bed so they don’t notice. Putting family first is what I always do.* (P 12)

#### Belief in the usefulness of non-scientific traditional methods

Some participants believed that some traditional medicines or drugs were very effective in reducing their symptoms and treating the disease, and thus used them. Among the traditional drugs, opium was the most common substance used by these patients. Participant number 7, who suffered from diabetes, said:

*I had high blood sugar for a while. I heard that opium can bring it down*,* so I tried it out. Turns out*,* it didn’t do anything. When I asked my doctor about it*,* he said it’s total nonsense and not a good idea at all.*

*I’m a big fan of traditional medicine*,* especially the stuff that opens up your arteries. I think it’s less harmful than those chemical drugs.* (P 11)

#### The taboo of expressing sexual problems

Sexual problems after heart failure were one of the problems mentioned by most of the participants and many questions were not asked due to shame and modesty and remained unanswered, causing this crucial care neglect.

*Some things are better left unsaid. Sexual problems can lead to emotional distance.* (P 1)

From the perspective of patients, the treatment team was also reluctant to discuss these issues. The cultural values and beliefs that dominate society may be the basis for this neglect.

*After I got sick*,* I started having problems in my relationship with my partner. I went to the doctor a bunch of times*,* but no one explained this issue to me. This is also a problem like other problems; they should tell us what to do about it.* (P 9)

### Weakening of mental-psychological power

According to the participants’ experience, they had neglected their care because of their diminished mental capacity. This category included the three subcategories of disappointment in the treatment process, fear of social stigma, and forgetting Gad.

#### Disappointment in the treatment process

Most of the participants had experienced a sense of hopelessness during the treatment process, which had caused them to become lax in taking care of themselves. This feeling was caused by their dissatisfaction with not the unfulfilled expectations of the treatments. The feeling of disappointment was expressed more by the patients who had been involved with the disease for a longer period of time. For example, Participant 3, who was born with a congenital heart defect, said:

*Sometimes I fed up with life. When I look back at all the hard times I’ve been through*,* I get discouraged. I didn’t have a good childhood. I was always in and out of the hospital. I’m sick*,* after all.* (P 3)

#### Fear of social stigma

In the experience of the participants, it was found that some of their self-care neglect is due to reasons such as fear of being judged and stigmatized by people.

*I said it would be better to get a cane. But with a walker it’s easier*,* less pressure on my heart; I’m embarrassed to use a walker*,* people think I’m paralyzed … I don’t like people seeing me like this.* (P 2)

*I don’t go to parties much; fatty party food isn’t good for me*,* but I eat in front of others. I don’t like it when people say that the poor person can’t eat.* (P 10)

#### Forgetting God

The participants’ statements showed that whenever they neglected the remembrance of God, their motivation for self-care decreased. They saw remembrance of God as a means of finding peace and relieving the stress of illness. Therefore, avoiding the remembrance of God and religious duties had led to their increased anxiety, decreased motivation, and self-care neglect.

*I’m more likely to neglect myself when I don’t think about God.* (P 2)

*Prayer gives me a lot of energy. When I’m lazy about praying*,* I feel worse. As my relationship with God gets weaker*,* I lose motivation to take care of myself.* (P 5)

### Synergy of physical problems

The participants’ experiences revealed that they suffer from many physical problems that cause them to neglect self-care. This category included two subcategories: Inability to stop bad eating habits and problems associated with comorbidities.

#### Inability to stop bad eating habits

The experience of the participants revealed that they do not have a proper adherence to the correct diet in the disease, which was attributed to the inability to change the wrong eating habits formed since childhood. Adding salt to food, consuming animal oils, drinking more fluids than allowed for heart patients, and consuming sugar were among the most important dietary habits reported by the participants. This issue can endanger their physical health and increase the risk of hypertension, hyperlipidemia, and obesity.

*I’ve always liked salty food. My parents always cooked with a lot of salt*,* so that’s what I’m used to. These diet foods are just not tasty to me. I need salt on the table*,* or I can’t eat anything.* (P 13).

#### Problems associated with comorbidities

Most participants suffered from several chronic diseases. All participants were taking a large number of daily medications prescribed by their physicians to treat their diseases. Taking this amount of medicine caused problems for them. These problems included forgetting to take medications, changes in medication dosages, medication interventions, medication side effects, and fear of these side effects. According to the participants, one of the reasons for their neglect of self-care was the presence of numerous diseases such as lung problems, arthritis, obesity, and the occurrence of complications caused by the disease, which limited their mobility, so that many of them did not have sufficient independence even in carrying out daily activities.

I take a lot of pills every day. Sometimes I forget which ones to take, because they all look the same. (P 2)

I’m in a lot of pains. I’ve severe arthritis too, and I had surgery on my knee. I need to walk for my heart, but I can’t because of my knee pain. (P 14)

### Inappropriate support

The participants felt that they had not received the support they needed after becoming ill. They stated that they did not receive the necessary support from the family, the government, and the healthcare system, indicating the improper condition for these patients. This category included the two subcategories of insufficient family support and insufficient government support.

#### Insufficient family support

Although the participants’ most important support systems were their family members, in cases where they had received inadequate family support, they neglected their self-care, because the role of family support is very important in advancing therapeutic goals.

*The doctor told me to go to the gym*,* but I don’t have anyone to watch my son. He’s naughty*,* and even my mom won’t take care of him.* (P 3)

#### Insufficient government support

As the participants mentioned, they had many problems in preparing their medicines because they did not receive any financial support from the government. Lack of financial support from the government and lack of support from the healthcare system were among the problems mentioned by all participants. Poor drug quality, inadequate insurance coverage, and high drug prices were among the issues that caused problems for patients. The participants also referred to the lack of free consultation centers for cardiac patients and the lack of modern equipment and technology. These factors have led to carelessness, such as fewer timely visits to the doctor, fewer visits for regular check-ups, and not taking or reducing the use of medications.

*Apparently it’s called insurance*,* but it doesn’t cover most of our treatment. Medicines aren’t covered either.* (P 6)

*My meds are so expensive! I don’t have any financial aid*,* and the government doesn’t do anything for us. I couldn’t afford to go to the doctor or buy my meds for a month*,* so I got worse and had to be hospitalized.* (P 15)

## Discussion

The present study was conducted to explain heart failure patients’ experiences of self-care neglect. The patients in their experiences of neglect in self-care pointed to false cultural beliefs in self-care, weakening of mental-psychological power, synergy of physical problems, and inappropriate support. The culture of any society has a direct effect on health and self-care during illness. Each culture contains certain views and beliefs that play an important role in shaping health-seeking behaviors and thus have crucial consequences for human health [27]. These beliefs include a wide range of values, norms, and taboos that may influence the decision to engage in self-care behaviors [27, 28]. The results of a study on diabetic patients in Ghana showed that false cultural beliefs, misconceptions, and stigmatization of patients by others were barriers to self-care behaviors [29]. In the present study, over-prioritizing family and prioritizing one’s spouse and children had a negative impact on the participants’ self-care. Iran is an ancient country with a rich and diverse culture and history. However, there is a strong focus on the family, the existence of strong family ties, and the importance of family members [30]. Although family ties provide support [31], paying too much attention to family and trying to care for them can cause patients to neglect themselves and their self-care needs. Instead of focusing on themselves, they spend all their energy taking care of their family members. The mass media and healthcare providers must improve the self-care for patients by creating a culture in this field.

Belief in the usefulness of non-scientific traditional methods was another false cultural beliefs mentioned by the participants. The use of traditional treatment methods exists in most cultures. These methods are used as a complement to modern treatments. However, the focus of this study was on non-scientific traditional methods that are often recommended by others and lead to self-care neglect. Despite the facts that some medicinal herbs have been used since ancient times to relieve some symptoms, their side effects and interactions with other drugs can have dangerous consequences in patients with polypharmacy [32]. Based on the results of a study, the prioritization of traditional treatments was one of the factors associated with poor medication adherence in cardiac patients [33]. Iranian culture has long held misconceptions about the magical effects of some herbal medicines and narcotics such as opium, which can lead to neglect of self-care. The taboo of expressing sexual problems caused by illness was one of the other cultural misconceptions in this study. In Iranian culture, talking about sexual issues is condemned and considered embarrassing, and by keeping this important need hidden, patients will face many problems. This cultural taboo also exists among medical personnel, which is considered a major obstacle to the lack of training in this area. Understanding these cultural beliefs is important for healthcare providers so that they can eradicate the behaviors and, while respecting the correct cultural values, discuss the patient’s cultural preferences, identify cultural misconceptions, and try to correct them.

In this study, another factor that influenced the patients’ self-care neglect was the weakening of the patients’ mental-psychological power, which was caused by the feeling of disappointment in the treatment process, fear of social stigma, and forgetting God. The results of a study indicated that stress, frustration, and depression can lead to changes in self-care and, as a result, worsen the patient’s heart disease [34]. Fear of social stigma was also a major barrier to a person’s participation in self-care and adherence to treatment [35]. The results of a systematic review showed a significant association between social stigma and cardiovascular health [36]. The results of a systematic review showed that adherence to spirituality and religious rituals in patients with heart disease was positively associated with medication adherence [30]. Therefore, feelings of hopelessness, neglect of religious duties, and disengagement from the remembrance of God, with a negative impact on mental health, can lead to self-care neglect. Therefore, it is recommended that for better results in the health of cardiovascular patients, mental healthcare should be done simultaneously with the care of the cardiovascular system.

Synergy of physical problems was another factor influencing patients’ neglect of self-care. The inability to stop bad eating habits, problems associated with polypharmacy, and restrictive physical problems were mentioned by the participants. A proper and healthy diet is one of the most important cares for heart patients [37]. Eating habits are formed in childhood and in the family environment [38], and changing these habits is very difficult for some people [29]. The inability to stop bad eating habits was described in this study as one of the important factors associated with negligence. Excess salt and sugar, harmful fats, and processed foods were among the dangerous eating habits of the participants. Due to the complex nature of the disease, people with cardiovascular disease tend to suffer from multiple conditions and take multiple medications. Approximately one-quarter of cardiac outpatients suffer from polypharmacy [39]. The risk of side effects increases with the amount of medication used. According to a study, poor medication adherence in cardiac patients may be caused by fear of dependence on multiple medications [33]. In this study, due to the number of medications, participants had lapses in medication adherence, such as forgetting a medication, changing the prescribed dose, or taking the wrong medication. Thus, it is necessary for healthcare providers to establish treatment goals for patients in order to manage their medication and periodically review the benefits and side effects of the medication. Moreover, patients and their family should be provided with the necessary education in the area of correct drug use and optimization of the prescribed medications.

Inappropriate support was another factor influencing patients’ neglect of self-care. Insufficient family support and insufficient government support were among the factors extracted from the experiences of the participants. A review of studies has indicated that chronically ill patients with more family support have higher levels of self-care [29, 40]. Self-care for chronic conditions such as cardiovascular disease requires support from family and society. The results of a study on heart patients showed that social support increased patients’ hope and improved their quality of life [41]. In the present study, poor drug quality, inadequate insurance coverage, high drug prices, and lack of necessary equipment and technology for patients had created many problems for the participants in the area of self-care. One study found that doubts about the efficacy of medications can also lead to poor medication adherence in cardiac patients [33]. Cardiovascular patients need regular diagnostic and therapeutic care, including visits to the doctor, frequent tests, and preparation of numerous drugs, which is very costly and many of these patients have severe financial problems. The government can support the care of cardiac patients by providing a comprehensive insurance system to manage the cost of drugs and treatment, and by establishing special centers with the most up-to-date facilities and free consultation services for cardiac patients.

### Strengths

This study was the first research on the experiences of patients with heart failure in the area of self-care neglect to be conducted in Iran. The results of this study may increase the knowledge of healthcare providers in the area of effective self-care support for these patients.

### Limitations

Given the fact that the participants of this study belonged to the Iranian culture and considering the differences between different cultures, it is suggested that future studies be conducted with more focus on cultural diversity in order to obtain more valid results.

## Conclusion

The patients in their experiences of neglect in self-care pointed to false cultural beliefs in self-care, weakening of mental-psychological power, synergy of physical problems, and inappropriate support. Preventing the occurrence of these factors and their correctionو can increase self-care capability and prevent side effects caused by neglect in these patients. Healthcare providers can help improve the health of these patients by designing interventions to reduce the desired factors. It is suggested that future research be designed as an intervention to reduce the effect of each of these factors.

## Data Availability

The datasets used and/or analysed during the current study are available from the corresponding author on reasonable request.

## Abbreviations

HF: Heart failure

## References

[1] Snipelisky D, Chaudhry SP, Stewart GC. The many faces of heart failure. Card Electrophysiol Clin. 2019;11(1):11–20. 30717842 10.1016/j.ccep.2018.11.001

[2] Wu J-R, Reilly CM, Holland J, Higgins M, Clark PC, Dunbar SB. Relationship of health literacy of heart failure patients and their family members on heart failure knowledge and self-care. J Fam Nurs. 2017;23:116–37. 28795936 10.1177/1074840716684808

[3] Javadi S, Salimi T, Anticchi M. The self-care behaviors in patients with heart failure admitted to Afshar Hospital in Yazd. Caring Today. 2017;9(32–33):1–12.

[4] Sinnenberg L, Givertz MM. Acute heart failure. Trends Cardiovasc Med. 2020;30(2):104–12. 31006522 10.1016/j.tcm.2019.03.007

[5] Tok Yildiz F, Kaşikçi M. Impact of Training based on Orem’s theory on Self-Care Agency and Quality of Life in patients with coronary artery disease. J Nurs Res. 2020;28(6):e125. 33017328 10.1097/JNR.0000000000000406 PMC7664957

[6] Navidian A, Moradgholi M, Kykhaee A, Saeedinegad F. Relationship between attachment styles and self-care behaviors in patients with heart failure. Hayat. 2015;21:6–17.

[7] Rosano G, Seferovic P, Strömberg A. Self-care of heart failure patients: practical management recommendations from the Heart Failure Association of the European Society of Cardiology. Eur J Heart Fail. 2021;23(1):157–74. 10.1002/ejhf.2008. 32945600 10.1002/ejhf.2008 PMC8048442

[8] Awoke MS, Baptiste DL, Davidson P, Roberts A, Dennison-Himmelfarb C. A quasi-experimental study examining a nurse-led education program to improve knowledge, self-care, and reduce readmission for individuals with heart failure. Contemp Nurse. 2019;55(1):15–26. 30632457 10.1080/10376178.2019.1568198

[9] Farghadani Z, Taheri-Kharameh Z, Airi-Mehra A, Montazeri A. Self-care behaviors and its related factors in patients with heart failure. Payesh. 2018;17(4):371–9.

[10] Jaarsma T, Cameron J, Riegel B, Stromberg A. Factors related to self-care in heart failure patients according to the Middle-Range Theory of Self-Care of Chronic Illness: a literature update. Curr Heart Fail Rep. 2017;14(2):71–7. 28213768 10.1007/s11897-017-0324-1 PMC5357484

[11] Asadi P, Ahmadi S, Abdi A, Shareef OH, Mohamadyari T, Miri J. Relationship between self-care behaviors and quality of life in patients with heart failure. Heliyon. 2019;5(9):e02493. 31687585 10.1016/j.heliyon.2019.e02493 PMC6819855

[12] Abe R, Sakata Y, Nochioka K, Miura M, Oikawa T, Kasahara S, Sato M, Aoyanagi H, Shiroto T, Sugimura K, Takahashi J, Miyata S, Shimokawa H. CHART-2 investigators. Gender differences in prognostic relevance of self-care behaviors on mortality and hospitalization in patients with heart failure - A report from the CHART-2 study. J Cardiol. 2019;73(5):370–8. 30606681 10.1016/j.jjcc.2018.11.006

[13] Nkabinde-Thamae G, Downing C, Nene S. Self-care neglect through the voices of nurses working in primary healthcare clinics in Gauteng, South Africa. Nurs Forum. 2022;57(6):1330–8. 36227150 10.1111/nuf.12812 PMC10092092

[14] https://www.oxfordlearnersdictionaries.com/definition/american_english/negligence

[15] Güler C, Engin E. A Self-Care Deficiency Syndrome: self-neglect. Psikiyatride Güncel Yaklaşımlar. 2023;15(4):622–30.

[16] Repetto EC, Zachariah R, Kumar A, Angheben A, Gobbi F, Anselmi M, et al. Neglect of a neglected disease in Italy: the challenge of Access-to-care for Chagas Disease in Bergamo Area. PLoS Negl Trop Dis. 2015;9(9):e0004103. 26406325 10.1371/journal.pntd.0004103 PMC4583374

[17] Day MR, Mulcahy H, Leahy-Warren P, Downey J. Self-neglect: a case study and implications for clinical practice. Br J Community Nurs. 2015;20(3):110. 25754778 10.12968/bjcn.2015.20.3.110

[18] Mansourieh N, Pour Sharifi H, Taban Sadeghi MR, Sirfi MR. The relationship between socioeconomic status and self-care in patients with heart failure: the role of illness related worries mediator. NPWJM. 2018;5(17):5–12.

[19] Victoria-Castro AM, Martin ML, Yamamoto Y, et al. Impact of Digital Health Technology on Quality of Life in patients with heart failure. JACC Heart Fail. 2024;12(2):336–48. 37943227 10.1016/j.jchf.2023.09.022

[20] Yoshinaga R, Tomita K, Wakayama K, Furuta S, Miyamoto K, Matsuda Y, et al. Factors related to self-care behaviors among hospitalized patients with heart failure in Japan, based on the European Heart failure self-care Behaviour Scale. J Phys Ther Sci. 2022;34(6):416–21. 35698558 10.1589/jpts.34.416 PMC9170480

[21] Vellone E, Fida R, Ghezzi V, D’Agostino F, Biagioli V, Paturzo M, et al. Patterns of self-care in adults with heart failure and their associations with Sociodemographic and clinical characteristics, Quality of Life, and hospitalizations: a cluster analysis. J Cardiovasc Nurs. 2017;32(2):180–9. 26938506 10.1097/JCN.0000000000000325

[22] Wu M, Peng C, Chen Y, Yuan M, Zhao M, Wang C, et al. Nurses’ perceptions of factors influencing elder self-neglect: a qualitative study. Asian Nurs Res. 2020;14(3):137–43. 10.1016/j.anr.2020.05.001 32603691

[23] Bengtsson M. How to plan and perform a qualitative study using content analysis. NursingPlus Open. 2019;2:8–14.

[24] Peters KA, Howe TJ, Rossiter D, Hutchinson KJ, Rosell PA. The abbreviated Mental Test score; is there a need for a contemporaneous update? Geriatr Orthop Surg Rehabil. 2021;12:21514593211001047. 34868721 10.1177/21514593211001047 PMC8634377

[25] Zhang Y, Wildemuth BM. Qualitative Analysis of Content. 2009. USA: Libraries Unlimited Inc; 520.

[26] Lincoln YS, Guba EG. Naturalistic Inquiry. Newbury Park: Sage; 1985.

[27] Hawley ST, Morris AM. Cultural challenges to engaging patients in shared decision making. Patient Educ Couns. 2017;100(1):18–24. 27461943 10.1016/j.pec.2016.07.008 PMC5164843

[28] Rahemi Z, Williams CL, Tappen RM, Engstrom GA. Health-related decisions for serious illness among ethnically diverse older adults. ANS Adv Nurs Sci. 2018;41(1):84–97. 29140805 10.1097/ANS.0000000000000192

[29] Mogre V, Johnson NA, Tzelepis F, Paul C. Barriers to diabetic self-care: a qualitative study of patients’ and healthcare providers’ perspectives. J Clin Nurs. 2019;28(11–12):2296–308. 30791160 10.1111/jocn.14835

[30] Elhag M, Awaisu A, Koenig HG, Mohamed Ibrahim MI. The Association between religiosity, spirituality, and medication adherence among patients with cardiovascular diseases: a systematic review of the literature. J Relig Health. 2022;61:3988–4027. 35274225 10.1007/s10943-022-01525-5 PMC9509306

[31] Osokpo O, Riegel B. Cultural factors influencing self-care by persons with cardiovascular disease: an integrative review. Int J Nurs Stud. 2021;116:103383. 31353026 10.1016/j.ijnurstu.2019.06.014

[32] Gouws C, Hamman JH. What are the dangers of drug interactions with herbal medicines? Expert Opin Drug Metab Toxicol. 2020;16(3):165–7. 32079422 10.1080/17425255.2020.1733969

[33] Belitsi V, Tsiampalis T, Kouvari M, Kalantzi V, Androutsos O, Bonoti F, et al. Exploring patient beliefs and medication adherence in the mediterranean context: a cross-sectional study in patients with cardiovascular diseases and cardiometabolic disorders in Greece—the IACT-Study. Life. 2023;13(9):1880. 37763284 10.3390/life13091880 PMC10532979

[34] Borkowski P, Borkowska N. Understanding Mental Health challenges in Cardiovascular Care. Cureus. 2024;16(2):e54402. 38505437 10.7759/cureus.54402 PMC10950038

[35] Stangl AL, Earnshaw VA, Logie CH, et al. The health stigma and discrimination framework: a global, crosscutting framework to inform research, intervention development, and policy on health-related stigmas. BMC Med. 2019;17:31. 30764826 10.1186/s12916-019-1271-3 PMC6376797

[36] Panza GA, Puhl RM, Taylor BA, Zaleski AL, Livingston J, Pescatello LS. Links between discrimination and cardiovascular health among socially stigmatized groups: a systematic review. PLoS ONE. 2019;14(6):e0217623. 31181102 10.1371/journal.pone.0217623 PMC6557496

[37] Esteban-Fernández A, Villar-Taibo R, Alejo M, Arroyo D, Bonilla Palomas JL, Cachero M, et al. Diagnosis and management of Malnutrition in patients with heart failure. J Clin Med. 2023;12(9):3320. 37176761 10.3390/jcm12093320 PMC10179706

[38] Kostecka M. Eating habits of preschool children and the risk of obesity, insulin resistance and metabolic syndrome in adults. Pak J Med Sci. 2014;30(6):1299–303. 25674127 10.12669/pjms.306.5792 PMC4320719

[39] Tefera YG, Alemayehu M, Mekonnen GB. Prevalence and determinants of polypharmacy in cardiovascular patients attending outpatient clinic in Ethiopia University Hospital. PLoS ONE. 2020;15(6):e0234000. 32479516 10.1371/journal.pone.0234000 PMC7263581

[40] Gul Zeren F, Canbolat O. The relationship between family support and the level of self-care in type 2 diabetes patients. Prim Care Diabetes. 2023;17(4):341–47. 37149410 10.1016/j.pcd.2023.04.008

[41] Soleimani MA, Zarabadi-pour S, Huak chan Y, Allen K, Shamsizadeh M. Factors associated with hope and quality of life in patients with coronary artery disease. J Nurs Res. 2022;30(2):e200. 35234211 10.1097/jnr.0000000000000476

